# Neoadjuvant docetaxel, oxaliplatin plus S-1 for treating clinical stage III squamous cell carcinoma of the esophagus: Study protocol of an open-label phase II trial

**DOI:** 10.1016/j.conctc.2021.100853

**Published:** 2021-11-09

**Authors:** Mitsuro Kanda, Dai Shimizu, Kazushi Miyata, Osamu Maeda, Chie Tanaka, Yoshikuni Inokawa, Norofumi Hattori, Masamichi Hayashi, Masahiko Ando, Yachiyo Kuwatsuka, Kenta Murotani, Goro Nakayama, Masahiko Koike, Yuichi Ando, Tomoki Ebata, Yasuhiro Kodera

**Affiliations:** a Department of Gastroenterological Surgery, Nagoya University Graduate School of Medicine, 65 Tsurumai-cho, Showa-ku, Nagoya, 466-8550, Japan; b Division of Surgical Oncology, Department of Surgery, Nagoya University Graduate School of Medicine, 65 Tsurumai-cho, Showa-ku, Nagoya, 466-8550, Japan; c Department of Clinical Oncology and Chemotherapy, Nagoya University Hospital, 65 Tsurumai-cho, Showa-ku, Nagoya, 466-8550, Japan; d Department of Advanced Medicine, Nagoya University Hospital, 65 Tsurumai-cho, Showa-ku, Nagoya, 466-8550, Japan; e Biostatistics Center, Graduate School of Medicine, Kurume University, 67 Asahi-cho, Kurume, 830-0011, Japan

**Keywords:** Esophageal cancer, Squamous cell carcinoma, Neoadjuvant chemotherapy, S-1, Clinical trial, ESCC, esophageal squamous cell carcinoma, DOS, docetaxel, oxaliplatin plus S-1, FP, 5-fluorouracil plus cisplatin, DCF, docetaxel, 5-fluorouracil plus cisplatin, RECIST, Response Evaluation Criteria in Solid Tumors

## Abstract

In Japan, esophagectomy after two courses of 5-fluorouracil plus cisplatin is regarded a standard strategy for treating resectable stage II or III esophageal squamous cell carcinoma (ESCC). However, 5-fluorouracil plus cisplatin does not benefit cohorts with clinical stage III ESCC, suggesting the requirement for a more effective regimen. We are conducting a single-arm phase II study to assess the safety and efficacy of neoadjuvant docetaxel, oxaliplatin plus S-1 (DOS) for treating patients with clinical stage III ESCC. The primary endpoint is the pathological response rate, and the target number is 45 patients. Safety, response rate, R0 resection rate, and survival are secondary endpoints. This trial is registered in the Japan Registry of Clinical Trials as jRCTs041210023. We are conducting a prospective phase II trial to evaluate the safety and efficacy of three courses of neoadjuvant DOS treatment followed by radical esophagectomy for clinical stage III ESCC.

## 1 Introduction

Esophageal squamous cell carcinoma (ESCC) is a dominant histological type of esophageal cancer in Asian countries [1]. Surgical resection contributes to prolonging the survival of patients with ESCC [[2], [3], [4]]. However, the high incidence of postoperative recurrence requires the administration of multidisciplinary treatment before or after surgery, or both [1,5].

In Japan, postoperative adjuvant chemotherapy was initially tested based on the concept that local control can be achieved through surgery and that systemic micrometastases should be controlled using systemic chemotherapy, with the expectation that such treatment would outperform surgery alone. The phase III JCOG9204 trial evaluated the survival benefit of postoperative administration of two courses of 5-fluorouracil plus cisplatin (FP) to 242 patients with pathological stage II-IV ESCC [6]. The trial found that postoperative administration of FP significantly increased the 5-year relapse-free survival rate (55%) compared with surgery alone (45%). However, there was no significant difference in 5-year overall survival. Meta-analysis did not reveal an overall survival benefit of postoperative chemotherapy vs surgery alone [7]. Therefore, insufficient evidence is available to support the conclusion that postoperative chemotherapy improves survival of resected patients with ESCC. However, the intolerance of patients to chemotherapy after esophagectomy likely explains the inability to achieve a sufficient relative dose intensity.

The justifications for using neoadjuvant chemotherapy include enhanced tolerability, increased curability through reduced tumor mass, and eradicability of micrometastasis [8]. To prove this concept, the phase III JCOG9907 trial was conducted to compare outcomes of neoadjuvant FP with those of postoperative adjuvant FP for stages II/III ESCC [9]. This trial found that neoadjuvant chemotherapy was superior for increasing overall survival compared with adjuvant chemotherapy (5-year overall survival 60% vs 38%, hazard ratio 0.64) [9]. Two courses of FP are therefore considered a standard treatment strategy for clinical stage II or III ESCC subsequent to esophagectomy.

Nonetheless, an important issue remains. Thus, the response rate to neoadjuvant therapy using FP in the JCOG9907 trial was 38%; and 2.5% of patients did not undergo surgery because of disease progression during neoadjuvant treatment [10]. Furthermore, subgroup analysis indicates that neoadjuvant therapy using FP conferred little survival benefit in the clinical stage III subgroup (hazard ratio, 0.94), in contrast to the clinical stage II subgroup (hazard ratio, 0.48) [10]. These results indicate that neoadjuvant treatment with stronger antitumor activity than that of FP is required to improve the prognosis of clinical stage III ESCC.

Docetaxel, 5-fluorouracil plus cisplatin (DCF) treatment was proposed as a more intensive triplet regimen. In a phase I clinical trial of patients with unresectable ESCC, 86% and 38% of 21 patients who received the recommended dose of DCF treatment had grade 3 or higher neutropenia and febrile neutropenia, respectively [11]. A phase II feasibility study in the neoadjuvant setting was performed for clinical stage II or III ESCC, except for T4. Grade 3 or higher neutropenia and febrile neutropenia were observed in 88% and 3% of patients, respectively, without treatment-related deaths [12]. The response rate was 62%, the R0 resection rate was 88%, and a pathological complete response was observed in 26% of patients [12]. A current phase III randomized clinical trial to compare the survival benefit of preoperative FP, preoperative DCF, and preoperative FP plus radiation therapy for clinical stage IB/II/III ESCC (JCOG1109) has completed enrollment and is undergoing prognostic follow-up [13].

Nevertheless, the DCF regimen may be toxic, requiring development of a regimen that is equally effective and less toxic in the setting of preoperative chemotherapy. For example, the oral fluoropyrimidine S-1 achieves efficacious concentrations of plasma 5-fluorouracil while reducing adverse gastrointestinal effects [14]. To test the safety and efficacy of S-1-based neoadjuvant treatment for clinical stage III ESCC, we previously performed a phase II clinical trial of two courses of neoadjuvant S-1 plus cisplatin [15]. Surgical resection was safely performed with an acceptable morbidity rate, and the 5-year progression-free survival and overall survival rates are 85% and 92%, respectively. The response rate based on endoscopy before the second course is 35%, which did not reach 55% as expected [15].

Two previous phase II clinical studies evaluated preoperative triplet chemotherapy including S-1 for ESCC. One trial tested three cycles of neoadjuvant docetaxel, cisplatin plus S-1, which achieved a 33% pathological response rate, in which 8% of 40 patients experienced febrile neutropenia [16]. The other trial used a neoadjuvant docetaxel, nedaplatin, and S-1 regimen that achieved a radiological response rate of 83%. Grade 3 or 4 neutropenia was experienced by 25% of 32 patients [17]. These studies suggest that S-1-based triplet chemotherapy is tolerable in the neoadjuvant setting for ESCC.

Docetaxel, oxaliplatin plus S-1 (DOS) treatment was developed with the expectation of a strong triplet effect and improved tolerability to oxaliplatin, which is less nephrotoxic than cisplatin. In addition, S-1 is less toxic than continuous administration of intravenous 5-fluorouracil. DOS is attractive as a preoperative chemotherapy, because clinical stage III ESCC requires a more intensive regimen than FP and can be administered to outpatients, because it does not require continuous hydration or continuous intravenous infusion. A multicenter phase III trial currently being conducted in South Korea evaluates the safety and efficacy of preoperative DOS treatment for cT3-4N0 or cT2-4N + gastric cancer (NCT01515748). The results will be published in the near future. The JCOG1704 phase II trial is being conducted in Japan to evaluate preoperative DOS treatment of advanced gastric cancer with extensive lymph node metastasis. However, we are unaware of clinical trials that assess the safety and efficacy of preoperative DOS treatment of patients with ESCC patients (jRCTs031180028).

In this context, we designed a prospective phase II trial to evaluate the safety and efficacy of three courses of neoadjuvant DOS treatment followed by radical esophagectomy for clinical stage III ESCC.

## 2 Methods

### 2.1 Ethics

The Nagoya University Certified Review Board approved the study protocol (number 2021-0068), which is registered in the Japan Registry of Clinical Trials (jRCT) as jRCTs041210023 (https://jrct.niph.go.jp/).

### 2.2 Objective

**Fig. 1:**
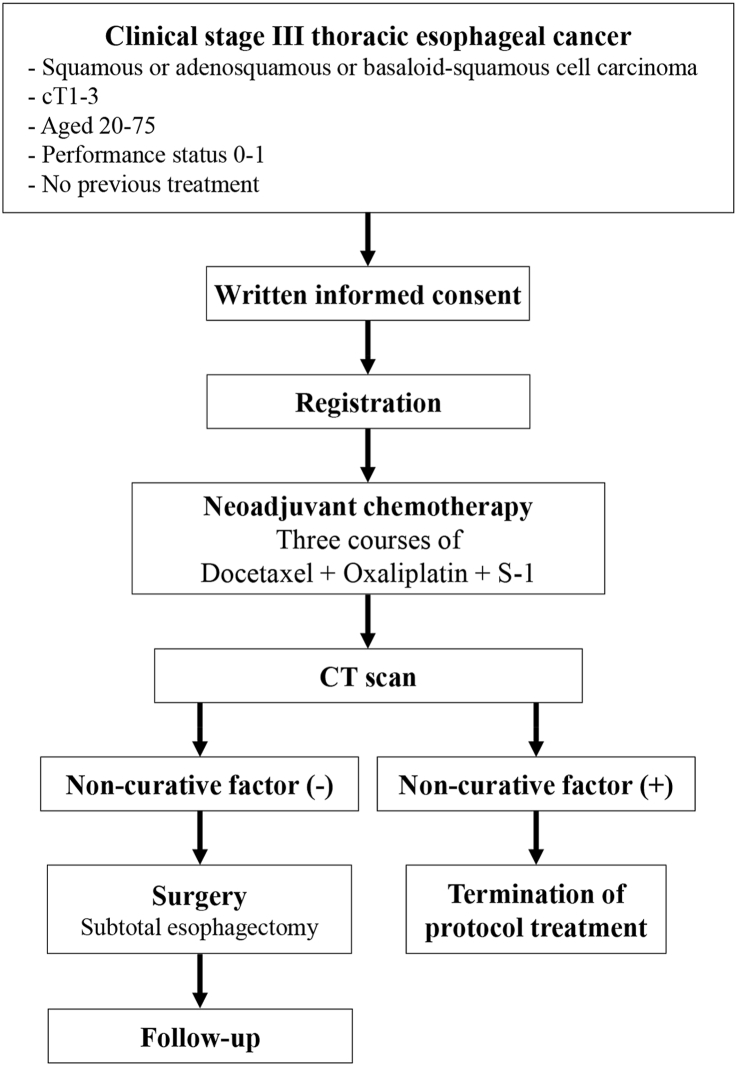
Protocol treatment.

To evaluate the safety and efficacy of treatment with neoadjuvant DOS for clinical stage III ESCC (Fig. 1).

### 2.3 Endpoints

The primary endpoint is the pathological response rate (grade 2 or 3). The pathological response is determined according to the proportion of viable cancer cells at the primary tumor and is defined as follows: grade 0, tumor unaffected; grade 1a, <one-third affected; grade 1b, one-third to two-thirds affected; grade 2, two-thirds to the entire tumor affected; and grade 3, undetectable viable cancer cells. Secondary endpoints are as follows: recurrence-free survival of patients who undergo R0 resection, overall survival, R0 resection rate, overall response rate (complete response or partial response) to neoadjuvant DOS according to the Response Evaluation Criteria in Solid Tumors (RECIST) ver. 1.1, treatment completion rate, and toxicity.

### 2.4 Eligibility criteria

#### 2.4.1 Inclusion criteria

Patients are enrolled when confirmed that they meet all of the eligibility criteria as follows:

i) Histologically confirmed esophageal squamous cell carcinoma, adenosquamous cell carcinoma, or basaloid squamous cell carcinoma
ii) Clinical stage III and T1-3 according to the 11th edition of the Japanese classification of esophageal cancer
iii) Main tumor located at the thoracic esophagus requiring subtotal esophagectomy
iv) Ages between 20 years and 75 years when informed consent was granted
v) Eastern Cooperative Oncology Group Performance Status 0 or 1
vi) No prior treatment
vii) Adequate function of vital organs, including bone marrow, liver, and kidney
viii) Written informed consent

#### 2.4.2 Exclusion criteria

i) Synchronous or metachronous (within 5 years) malignancies, except for carcinoma in situ
ii) Active infectious disease
iii) Psychiatric disease
iv) Continuous systemic steroid therapy or warfarin
v) Active interstitial lung disease or pulmonary fibrosis
vi) Active heart failure or ischemic cardiac disease
vii) Persistent intestinal bleeding
viii) Allergy to docetaxel, oxaliplatin, or S-1
ix) Positive HBs antigen
x) Ineligible by physician's assessment

### 2.5 Registration

A registration form will be sent to the registration center at the Chubu Clinical Oncology Group (CCOG) after written informed consent is obtained.

### 2.6 Treatment procedure

**Fig. 2:**
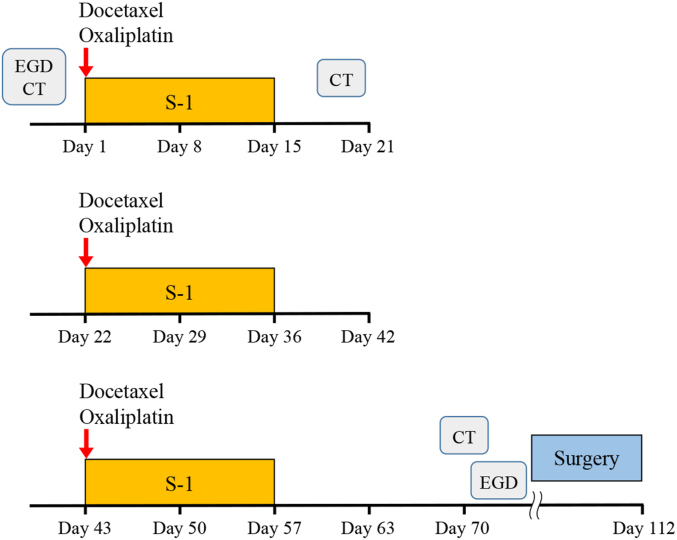
Trial design.

Three cycles of DOS are administered within 14 days after enrollment. S-1 is orally administered twice daily for the first 2 weeks of a 3-week cycle. The dose of S-1 administered at each time is calculated according to the patient's body surface area as follows: >1.25 m^2^, 40 mg; 1.25–1.5 m^2^, 50 mg; and >1.5 m^2^, 60 mg. Docetaxel (40 mg/m^2^) and oxaliplatin (100 mg/m^2^) are intravenously administered on day 1 of each cycle. Patients are scheduled for surgery within 56 days after the last dose of chemotherapy (Fig. 2).

Each drug dose is reduced according to the study protocol when patients exhibit adverse effects. The study protocol includes detailed algorithms for managing drug-specific toxicities such as S-1-related diarrhea and oxaliplatin-related peripheral neuropathy as well as other treatment-related toxicities. Toxicities are evaluated during chemotherapy according to laboratory (blood and urine) tests and physical examinations, and grading is determined according to the criteria of the National Cancer Institute “Common Terminology for Adverse Events” (version 5.0). Briefly, dose reduction or drug cessation are determined when patients experience grade-3 nonhematological toxicity, hematological toxicity (grade 4 neutropenia, grade 3 febrile neutropenia, or both, or ≥ grade-3 decrease in platelets).

Patients undergo subtotal esophagectomy with systematic lymphadenectomy (2- or 3-fields) through a thoracoscopic or right thoracotomy approach within 56 days after the last date of S-1 administration. The reconstruction method is selected according to the surgeon's discretion or considering patient's condition. Postoperative adjuvant treatment before disease recurrences is not permitted.

### 2.7 Follow-up

CT scans of the cervical area, chest, and abdomen as well as upper gastrointestinal endoscopy are performed within 28 days before enrollment. CT scans to determine the response to treatment are performed within 7 days before initiation of the second course of neoadjuvant DOS and before surgery. The levels of serum tumor markers (CEA and SCC-Ag) are determined before enrollment and surgery.

After protocol treatment, patients are followed every 3 months during the first postoperative year and then every 6 months for ≥2 years. Blood tests, including serum levels of SCC-Ag and CEA, and physical examinations are performed at every scheduled visit. Upper gastrointestinal endoscopy is performed once each year. Contrast-CT scans of the cervical, chest, and abdominal regions are performed every 6 months for 3 years after surgery and once every year thereafter. Treatment after disease recurrence is not prescribed.

### 2.8 Sample size plan

A study by Kelson et al. and the JCOG9907 trial reported that the pathological response rates to neoadjuvant 5-fluorouracil plus cisplatin treatment are 20% and 23% respectively [9,18]. We accordingly set threshold and expected response rates of 25% and 45%, respectively. When we determined a significance level = 95%, α-error = 0.05, and β-error = 0.2 (one-sided), we required 41 patients to detect statistically significant differences according to a calculation using the One Sample Binomial method of the Southwest Oncology Group (target sample size of 45), with an estimation that approximately 20% of the final subject population will be lost. The planned registration period is three years.

### 2.9 Statistical analyses

Statistical analysis upon completion of the trial will be performed using SAS version 14.2 software (SAS Institute, Cary, NC). We will use the Cox proportional hazards model to calculate hazard ratios and confidence intervals and the Kaplan–Meier method to analyze survival curves. Overall survival is defined as the date of enrollment until the date of death from any cause. Recurrence-free survival is defined as the interval from surgery to the date of the first documented detection of a recurrence.

## Funding

This work was supported by 10.13039/501100014807Nagoya University Hospital Funding for Clinical Development.

## Trial registration

The Japan Registry of Clinical Trials (jRCT) jRCTs041210023. Registered on May 21, 2021. (https://jrct.niph.go.jp/).

## Declaration of competing interest

The authors declare the following financial interests/personal relationships which may be considered as potential competing interests: Masahiko Ando: Grant from 10.13039/501100004095Kyowa Kirin Co. Ltd., outside the submitted work.

Yasuhiro Kodera: 10.13039/100010795Chugai Pharma (Inst), Daiichi Sankyo (Inst), 10.13039/100009954Taiho Pharmaceutical (Inst), 10.13039/100008373Takeda (Inst), Abbott (Inst), 10.13039/100004339Sanofi (Inst), 10.13039/501100012030Yakult (Inst), Eli 10.13039/100004312Lilly (Inst), 10.13039/100004319Pfizer (Inst), 10.13039/501100013170Ono Pharmaceutical (Inst), Kaken Pharmaceutical (Inst), 10.13039/501100013420Tsumura (Inst), 10.13039/501100002736Covidien (Inst), 10.13039/100014421EA Pharma (Inst), Otsuka (Inst), Nippon Kayaku (Inst), 10.13039/501100007132Otsuka Pharmaceutical Factory (Inst), 10.13039/100004331Johnson & Johnson K.K. (Inst), Sawai Pharmaceutical (Inst), 10.13039/501100005612Shionogi and MSD (Inst) (RF); 10.13039/100010795Chugai Pharma, Eli 10.13039/100004312Lilly, 10.13039/100004331Johnson & Johnson, 10.13039/501100012030Yakult Honsha, 10.13039/100009954Taiho Pharmaceutical, 10.13039/501100013170Ono Pharmaceutical, Medtronic, MSD, 10.13039/100010477Intuitive Surgical, Miyalisan Pharmaceutical, Nippon Kayaku, Daiichi Sankyo, Otsuka, Sawai Pharmaceutical, and 10.13039/501100013420Tsumura (H). (RF) Research funding; (H) Honoraria received.

Yuichi Ando: Dr. Ando reports grants and personal fees from 10.13039/100010795Chugai Pharmaceutical Co., Ltd., grants and personal fees from 10.13039/501100004095Kyowa Kirin Co., Ltd., grants and personal fees from Nippon Kayaku Co., Ltd., grants and personal fees from 10.13039/501100012030Yakult Honsha Co., Ltd., personal fees from 10.13039/100014422Eli Lilly Japan K.K., grants from Mochida Pharmaceutical Co., Ltd., grants and personal fees from 10.13039/501100013170Ono Pharmaceutical Co., Ltd., grants and personal fees from 10.13039/100009954Taiho Pharmaceutical Co., Ltd., personal fees from 10.13039/100008792Novartis Pharma K.K., personal fees from 10.13039/100004326Bayer Holding Ltd., personal fees from 10.13039/100002491Bristol-Myers Squibb, personal fees from Sawai Pharmaceutical Co., Ltd, grants and personal fees from 10.13039/501100002336Daiichi Sankyo Company, Ltd., grants and personal fees from 10.13039/501100003769Eisai Co., Ltd., personal fees from 10.13039/501100013420Tsumura & Co., personal fees from Otsuka Holdings Co., Ltd., personal fees from 10.13039/100016545Roche Diagnostics K.K., personal fees from 10.13039/100004325AstraZeneca K.K., personal fees from MSD K.K, outside the submitted work.

The other Authors indicated no financial relationships.
